# Comparison of whole-body diffusion-weighted magnetic resonance and FDG-PET/CT in the assessment of Hodgkin’s lymphoma for staging and treatment response

**DOI:** 10.3332/ecancer.2014.429

**Published:** 2014-05-15

**Authors:** Juan Montoro, Daniele Laszlo, Natalia Pin Chuen Zing, Giuseppe Petralia, Giorgio Conte, Marzia Colandrea, Giovanni Martinelli, Lorenzo Preda

**Affiliations:** 1 Division of Clinical Haematology/Oncology, European Institute of Oncology, Via Ripamonti 435, Milan 20141, Italy; 2 Division of Radiology, European Institute of Oncology, Via Ripamonti 435, Milan 20141, Italy; 3 Department of Health Science, University of Milan, Milan 20216, Italy; 4 Division of Nuclear Medicine, European Institute of Oncology, Via Ripamonti 435, Milan 20141, Italy

**Keywords:** Hodgkin’s lymphoma, PET/CT, whole-body diffusion-weighted magnetic resonance

## Abstract

Computed tomography (CT), 18F-fluorodeoxyglucose positron emission tomography (FDG-PET), and hybrid FDG-PET/CT are the most commonly used diagnostic tools for the initial staging and treatment response assessment of lymphomas [1]. The aim of this report is to compare the correlations between functional imaging markers derived from FDG-PET/CT and whole-body, diffusion-weighted magnetic resonance imaging (DW-MRI) in a young patient affected by Hodgkin’s lymphoma (HL).

We report a case of a nodular sclerosis HL with a bulky presentation at diagnosis in a 20-year-old female patient, who underwent FDG-PET/ CT and whole-body DW-MRI before and after autologous peripheral stem cell transplantation (APSCT). The patient received a standard chemotherapy regimen of adriamycin, bleomycin, vinblastine, and dacarbazine for six cycles, and the response was consolidated with APSCT. PET/CT images were obtained starting 50 min after the administration of the 18F-FDG i.v. injection (3.5 MBq/kg), from skull base to pelvis (3 min, bed position). FDG-PET/CT response evaluation was assessed according to the Deauville criteria [2] using a five-point scale in which the preferable reference scale was the mediastinum and the liver. Whole-body DW-MRI included both DW sequence (21 min and 52 s) and morphological sequences without contrast agent administration performed at 1.5T (Avanto, Siemens), from head to pelvis, with a cumulative acquisition time of 41 min and 7 s. Both FDG-PET/CT and whole-body DW-MRI performed before treatment showed advanced-stage disease (Ann Arbor stage IV): bulky nodal disease above and below the diaphragm, extensive hepatic and splenic disease, and multiple bone lesions (Figure 1a and b). The patient received a standard chemotherapy regimen and the response was consolidated with high-dose therapy and autologous stem cell transplant. Both post-treatment imaging examinations performed six months later demonstrated a complete remission (Figure 2a and b). Conventional radiology and CT are generally unable to detect the differences between tumour tissue and fibrosis [3]. To date, FDG-PET/CT has proven to be useful in the evaluation and management of HL patients, and it is currently the state-of-the-art imaging technique for staging, restaging, and response assessment at the end of treatment [4].

There are limitations for FDG\PT, for example, the non-detection of low-grade histologic subtype (extranodal marginal zone, small lymphocytic lymphoma/chronic lymphocytic leukaemia, and mycosis fungoides), and minimal residual disease under the spatial resolution of the device (4–5 mm). On the other hand FDG\PT detects benign conditions with increased glycolysis (such as infection, inflammation, granulomatous disease, and bone marrow hyperplasia), and high physiological uptake in brown adipose tissue, which may obscure or mimic the presence of tumour deposits. Furthermore, there is the necessary exposure of the patient to ionizing radiation [5]. Whole-body DW-MRI may represent an excellent alternative providing a combination of anatomical and functional information, useful for determining both the exact location and the extent of the abnormality, as well as possible tumour activity [6]. DW-MRI explores the random (Brownian) motion of water molecules in biological tissues and allows the calculation of an apparent diffusion coefficient (ADC), a quantitative parameter. ADC provides information concerning cellularity, which is significantly higher in many malignant tumours, including lymphomas, than in benign, and normal tissues [7, 8].

Whole-body DW-MRI is well tolerated by patients thanks to the relatively shorter acquisition time, which is longer than that for total body CT but shorter than that for PET/CT, and the absence of the need for contrast agent administration and radiation, which are particularly relevant for young patients requiring repeated, follow-up surveillance. Furthermore, the lack of contrast agent administration makes whole-body DW-MRI particularly suited for patients with renal function impairment, or that are allergic to iodinated contrast agents. The principal limitations of whole-body DW-MRI include the exclusion of patients with pacemakers, implantable defibrillators or debilitating claustrophobia, and the limited nationwide availability of adequate MR scanners [8]. This article shows the value of this MRI technique not only for staging, but also for the assessment of response in HL. We suggest that DW-MRI and FDG-PET/CT may be equivalent imaging modalities. DW-MRI represents an alternative procedure during staging and treatment response in HL patients, although further studies are needed to definitively introduce this diagnostic modality in the current standard of care.

## Disclosure of conflict of interest

The authors declare that they have no conflict of interest.

## Figures and Tables

**Figure 1:**
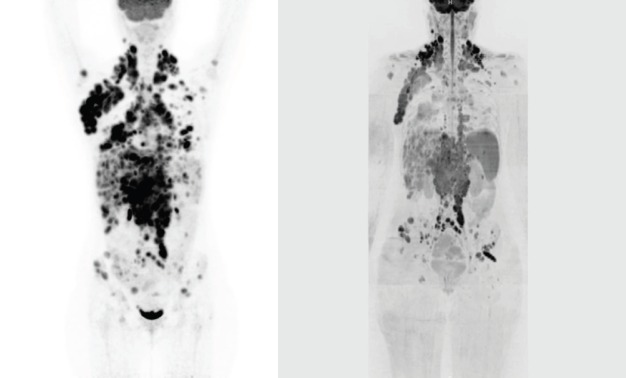
(a) Coronal maximum intensity projection (MIP) FDG-PET/CT performed at initial staging showing right axillary, bilateral cervical, mediastinal, lombo-aortic and pelvic lymph node involvement, multiple hepatic lesions, splenomegaly, humeral, vertebral, and pelvic bone marrow involvement. (b) Coronal, whole-body, grayscale inverted MIP DW-MRI performed at initial staging showing extensive disease with the same distributions as FDG-PET/CT.

**Figure 2:**
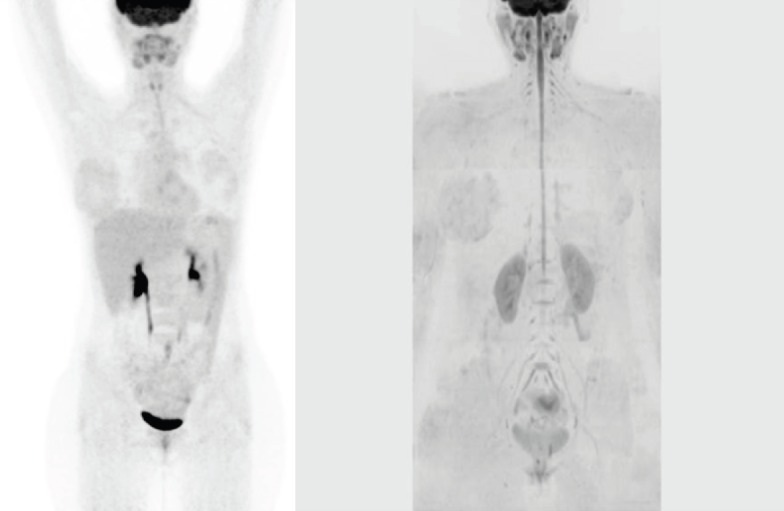
(a) Coronal MIP FDG-PET/CT performed six months after treatment demonstrating the absence of any residual abnormal activity. (b) Post-treatment coronal, whole-body grayscale inverted MIP DW-MRI showing no nodal or extranodalresidual disease.
